# Action video game play and transfer of navigation and spatial cognition skills in adolescents who are blind

**DOI:** 10.3389/fnhum.2014.00133

**Published:** 2014-03-11

**Authors:** Erin C. Connors, Elizabeth R. Chrastil, Jaime Sánchez, Lotfi B. Merabet

**Affiliations:** ^1^The Laboratory for Visual Neuroplasticity, Department of Ophthalmology, Massachusetts Eye and Ear Infirmary, Harvard Medical SchoolBoston, MA, USA; ^2^Department of Psychological and Brain Sciences, Center for Memory and Brain, Boston UniversityBoston, MA, USA; ^3^Department of Computer Science, Center for Advanced Research in Education, University of ChileSantiago, Chile

**Keywords:** early blind, adolescent, navigation, spatial cognition, gaming for learning, serious videogames, virtual environment

## Abstract

For individuals who are blind, navigating independently in an unfamiliar environment represents a considerable challenge. Inspired by the rising popularity of video games, we have developed a novel approach to train navigation and spatial cognition skills in adolescents who are blind. Audio-based Environment Simulator (AbES) is a software application that allows for the virtual exploration of an existing building set in an action video game metaphor. Using this ludic-based approach to learning, we investigated the ability and efficacy of adolescents with early onset blindness to acquire spatial information gained from the exploration of a target virtual indoor environment. Following game play, participants were assessed on their ability to transfer and mentally manipulate acquired spatial information on a set of navigation tasks carried out in the real environment. Success in transfer of navigation skill performance was markedly high suggesting that interacting with AbES leads to the generation of an accurate spatial mental representation. Furthermore, there was a positive correlation between success in game play and navigation task performance. The role of virtual environments and gaming in the development of mental spatial representations is also discussed. We conclude that this game based learning approach can facilitate the transfer of spatial knowledge and further, can be used by individuals who are blind for the purposes of navigation in real-world environments.

## INTRODUCTION

Navigating successfully in an unfamiliar environment represents a considerable challenge for individuals who are blind. In the absence of sight, they must rely on non-visual cues to gather sensory information and create a mental spatial representation of their surroundings (Strelow, 1985; Ashmead et al., 1989; Loomis et al., 1993). To remain functionally independent and learn important navigation skills, individuals who are blind typically receive formal training in orientation and mobility (O&M; Blasch et al., 1997; Thinus-Blanc and Gaunet, 1997). Despite this training, learning the spatial layout of a large scale complex environment and developing strategies to promote the mental manipulation of spatial information (for example, to devise an alternate route or find a shortcut for more efficient travel) remain difficult skills to master, particularly in younger individuals with early onset blindness (Cornoldi et al., 1979, 2009; Vecchi et al., 2004). A number of novel assistive technologies and electronic travel aids (such as sensory canes, GPS based travel aids, and sensory substitution devices) have been developed to facilitate navigation (Petrie et al., 1996; Loomis et al., 2005; Johnson and Higgins, 2006; Giudice et al., 2007; Kalia et al., 2010; Chebat et al., 2011; see also Roentgen et al., 2012 for a comparative evaluation of assistive devices for navigation). However, it is important to note that typically less emphasis has been placed on using assistive devices for the specific purpose of training spatial cognitive skills that are crucial to promote more efficient travel and independent navigation [for examples of studies in the effort, see (Giudice and Tietz, 2008; Giudice et al., 2010)]. The combination of virtual environments and video games represents a potentially exciting and novel opportunity to motivate learning and supplement traditional training strategies (Shaffer et al., 2005; Deubel, 2006; Dede, 2009; Spence and Feng, 2010; Bavelier et al., 2011). In the case of blindness, interaction through non-visual virtual environments have been used as a means to teach important concepts and subject material (such as problem solving in science and mathematics) as well as interacting with complex spatial constructs that might otherwise be difficult to learn through more traditional didactic means (Sánchez and Lumbreras, 1998; Lahav, 2006; Sánchez and Saenz, 2006; Sánchez and Maureira, 2007; Sánchez et al., 2009; Afonso et al., 2010; Saenz and Sánchez, 2010; Lahav et al., 2012; Lange et al., 2012; Merabet et al., 2012)

With the specific goal of promoting navigation skills in individuals who are blind, we have developed Audio-based Environment Simulator (AbES). This software represents a virtual rendering of an existing physical building that can be explored using audio cues alone. Specifically, the purpose of the virtual environment is to allow for the “offline” survey of a given spatial layout prior to navigating in the corresponding physical environment represented. Using a keyboard and simple key strokes, a user navigates through a target virtual environment acquiring contextually relevant spatial information in a manner that allows the individual to generate a mental representation of a building’s layout. To further promote self-directed and full exploration of the virtual environment, a game based approach is employed in which the user engages in goal-directed, action video game metaphor. The underlying game metaphor requires the user to search for randomly hidden jewels, remove them from the building, and avoid roving monsters that are programmed to take away the jewels and hide them in new locations.

As part of a larger scale programmatic study investigating the use of virtual environments to teach navigation skills in individuals who are blind, we have previously reported (as a proof of concept) that the immersive and highly interactive nature of AbES greatly engages blind users to actively explore a target virtual environment (Merabet et al., 2012). Furthermore, interacting with AbES within the context of a video game metaphor (as opposed to a more structured and didactic path learning approach) appears to facilitate the learning and transfer of navigation skills when assessed in the target building represented in the software (Merabet et al., 2012). As part of these preliminary findings, we observed that a subset of individuals, namely adolescents with blindness of early onset and who are self-reported strong users of technology, appeared to show higher levels of performance when using this game based training approach as compared to older participants.

Based on these initial findings, we now conducted a focused study in a targeted population of interest, i.e., early blind adolescents. This study had two primary goals. First, to validate whether learning the spatial layout of a building through video game play (using AbES) would lead to the direct transfer of spatial cognitive skills as evidenced by a set of navigation task assessments carried out in the real-world building modeled in the game. These navigation tasks were designed to assess participants’ ability to transfer and mentally manipulate the spatial information acquired from game play. Second, we wished to determine whether the transfer of spatial skills related to navigation task performance was correlated with participants’ success in game play.

## MATERIALS AND METHODS

### AbES SOFTWARE DESIGN

Employing a user-centered design and drawing input from potential end-users (i.e., conversations, interviews, and usability evaluations), we developed AbES as a virtual environment simulator based on the floor plans and spatial layout of a real physical building that can be explored and navigated through non-visual means. The rendering of the virtual environment consists of different structural elements (e.g., walls, stairs, rooms) as well as objects (e.g., doors, desks, and tables). A user can freely explore the environment for the purposes of gaining familiarity with the building’s overall layout. The software was developed using C++ with Visual Studio.NET and framework 2.0 using a PC computer (Windows XP/7 operating system) and can be run using a 10Mb HD, 1Gb RAM Pentium processor or higher. For the purposes of this study, a 2.2 GHz Intel Pentium Dual Core processor was used. Audio verbal commands and iconic sounds were pre-recorded and stored as sound files. The interfaces defined for AbES correspond to audio, graphic and keyboard formats. The spatial audio system allows the user to identify their egocentric heading, position and orientation, and the relative location of the objects found in the building. User interaction is carried out using the computer keyboard and specific keys for actions such as moving, opening doors, or interacting with objects (for further details, see the following section). The graphic interface represents an abstraction of the state of the game, showing the building layout and the location of objects and characters that are involved with game play. The graphic interface is used by the investigator or training facilitator and has configuration settings (e.g., number of jewels and monsters) and performance metrics that can be collected for further analysis.

### STUDY PARTICIPANTS, TRAINING, AND GAME PLAY

Seven early blind adolescents aged between 16 and 17 years participated in the study (three males; all with documented blindness prior to the age of three and familiar with the use of a computer keyboard interface; see **Table 1**). All provided written informed consent in accordance with procedures approved by the investigative review board of the Massachusetts Eye and Ear Infirmary (Boston, MA, USA). Training and performance assessments were carried out at the Carroll Center for the Blind (Newton, MA, USA). All participants were determined to be as strong users of technology based on their response to a questionnaire collected prior to game play. Specifically, we asked: “Using a 4-point scale (where 1 signifies very little, and 4 signifies very much/extensive), how would you rate your use of technology and electronic devices such as a smart phone, computer, email and texting, and entertainment devices such as an iPod?” The mean score for this question item was 3.29 ± 0.49 SD suggesting that on average, participants were relatively strong users of technology.

**Table 1:** Participant demographics.

ID	Age (years)	Gender	O&M experience (years)	Cause of blindness
1	17	Female	14	Retinopathy of prematurity
2	16	Female	1	Glaucoma
3	16	Female	13	Ocular albinism
4	17	Male	14	Bilateral anophthalmia
5	17	Male	3	Posterior polymorphous dystrophy
6	16	Male	6	Familial exudative vitreo-retinopathy
7	17	Female	1	Complications due to infection

Training included 2, 30-min sessions with an initial familiarization period (roughly 15 min) to learn the rules and key strokes for game play. All study participants were previously unfamiliar with the target building layout as well as naïve to the overall purpose of the study so as to minimize any potential confounds related to prior familiarity and expectation bias. Furthermore, at no time during the training were the participants instructed to keep track of the overall layout of the building, nor were they told that they would be eventually tested on their navigation ability. In this manner, we assumed that navigation performance in the target building without any previous training would be functionally at floor.

**FIGURE 1:**
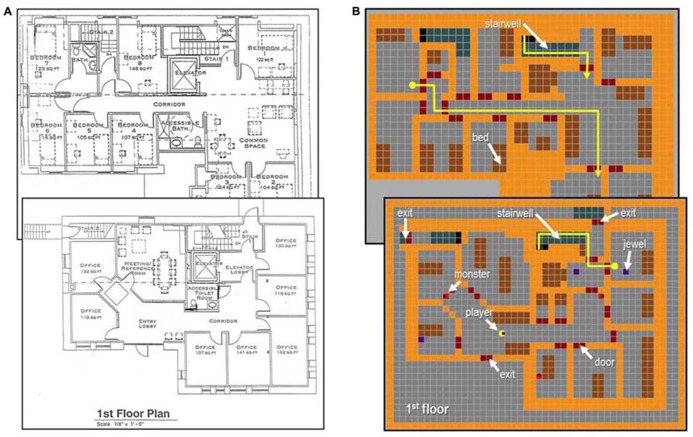
**Virtual rendering of a physical environment represented in the AbES software.** **(A)** architectural floor plan of an existing two story building with 23 rooms, two stairwells and three exits (first floor is shown in the foreground). **(B)** virtual rendering of the same building and set up for self-exploration in the context of a game metaphor. The player (yellow icon) navigates through the virtual environment using auditory cues to locate hidden jewels (blue squares) and avoid being caught by chasing monsters (red icons). Two example routes used to assess navigation performance are shown in yellow (start: round circle, end: arrow head).

All participants wore a blindfold throughout the training and assessment period (to eliminate any potential confounding effects relates to residual visual function) and used peri-auricular designed stereo headphones to listen to the various auditory spatial cues while interacting with the AbES software. The virtual environment rendered in AbES represents an accurate and to scale representation of an existing two-story building (23 rooms, two stairwells, three exits; **Figures 1A,B**). The design details of the AbES software have been described in detail previously [see (Sánchez et al., 2009, 2010a,b)]. Briefly, as the user navigates through the virtual building, they sequentially acquire updated auditory-based and contextual spatial information. The spatial information is based on iconic and spatialized sound cues provided after each step taken. The software is designed to play an appropriate audio file as a function of the location and egocentric heading of the user, and keeps track of the user’s position as they move through the environment. For example, if a door is located on the person’s right side, the knocking sound is heard in the user’s right ear. If the person now turns 180° so that the same door is now located on their left side, the same knocking sound is now heard in the left channel. Finally, if the user is facing the door, the same knocking sound is heard in both ears equally. Orientation is based on cardinal compass headings (e.g., “north” or “east”) and text to speech (TTS) is used to provide further information regarding a user’s current location, orientation and heading (e.g., “you are in the corridor, on the first floor, facing west”) as well as the identity of objects and obstacles in their path (e.g., “this is a door”). Distance cues are provided based on modulating sound intensity (e.g., the alert sound of a nearby jewel increases as the user approaches and the sequence of steps taken in a stairwell increases in pitch as user climbs up the stairs).

By keeping track of the user’s egocentric heading, the software plays the appropriate spatially localized sounds that identify the presence and location of objects and keeps track of these changes as the user moves through the virtual environment. It is important to note that TTS information did not provide any direction indices or step-by-step instructions to reach a target. Rather, it served as only ancillary information such as to identify the name of a particular room, identify an object, or provide heading information as needed. Thus, the virtual environment was designed to make maximal use of the spatial audio cues provided as a primary means of acquiring contextual information regarding the environment’s spatial layout. The audio information was meant to be sufficient to allow a user to explore the environment in a self-directed manner and without further assistance from a facilitator.

Using key strokes (e.g., “H” to turn left, “K” to turn right, “J” to open doors, “F” to identify current location, and “space bar” to move forward), participants were encouraged to explore the virtual environment and search for as many randomly placed jewels as possible in the allotted time (total of 60 min). Upon collecting a jewel, the participants were instructed to remove it from the building using one of the three possible building exits before securing another jewel (see **Figure 1B**). Participants also had to avoid roving monsters programmed to take away the jewels and hide them in new locations. This latter design feature was implemented in order to promote the user’s continuous search and exploration of the building including areas that were previously encountered.

### ASSESSMENT OF BEHAVIORAL PERFORMANCE (GAME PLAY AND NAVIGATION)

As an index of game play performance, we recorded the number of “jewel points” collected in the 60 min period. For each successful jewel removed, a corresponding point value was assigned (from 0 to 3) that reflected the path the participant used to exit the building. Specifically, 3 points were given for using the closest possible exit from where the jewel was found, 2 points for the next closest, 1 point for the farthest, and 0 points if they were unable to find an exit and remove the jewel. In this manner, this index measure characterizes the participant’s ability to acquire, update, and actively manipulate their mental spatial representation of the building layout (in-line with the premise of the game and overall goal for training). Since participants encountered jewels largely by chance, the comparative raw count of number of jewels collected did not sufficiently characterize the path strategy chosen, nor accurately reflect the user’s ability to manipulate their mental representation of the building layout.

Following game play, participants were taken to the physical building modeled in the AbES software and navigation performance was assessed with two behavioral tasks. In task 1 (direct route finding), navigation performance was evaluated on a series of 10 randomly presented pre-determined paths of comparable length and complexity (i.e., distance traveled and number of turns, see **Figure 1B**). Specifically, the range of steps needed to navigate the target route ranged between 25 and 35 steps and incorporated between 3 and 5 90° turns. Participants were brought to a start point (by a sighted experimenter) and instructed to navigate to a pre-specified target location (i.e., another room in the building). The start and end points could be located on the same floor or separate floors, requiring the participant to use the stairwells to reach the target. Navigation success was assessed by the number of correct paths executed (expressed as mean percentage correct). A maximum time limit of 6 min was allowed for each path attempted. While there was no direct measure of chance performance, it is important to note that there were 23 possible target rooms from any given starting point (including returning to start) and that scoring was based on the participants’ first verbal response upon arrival to a target. In this way, participants were not able to indicate every possible destination within the time allotted in the hopes of finding the correct target at random.

**FIGURE 2:**
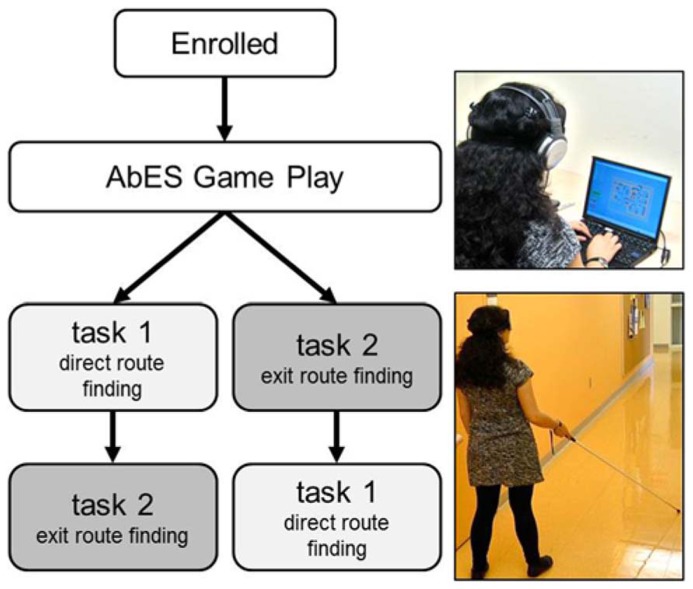
**Study Design.** Once enrolled, all eligible participants underwent the same training and game play period. Following game play, two navigation assessments (10 trials each) were carried out and the order of assessment was randomized across participants.

In task 2 (exit route finding), participants were brought to a series of 10 randomly presented, predetermined starting points and instructed to exit the building (i.e., using one of the three possible exits). Similarly, performance success was assessed by the number of correct paths executed (expressed as mean percentage correct). Again, a maximum time of 6 min was allowed for each path attempted. For each starting point, there was one optimal (i.e., shortest) possible path. Thus as a secondary measure, we also quantified the number of shortest paths carried out by the participant (expressed as mean percentage of shortest paths selected). To minimize the potential of a learning effect carrying over from sequential task assessment, a counterbalanced design was employed (**Figure 2**).

Planned measures of association between the main factor of interest (i.e., jewel points accrued during training) and navigation performance in the two tasks were calculated using the Pearson product-moment correlation coefficient (following confirmed tests for normality). Secondly, as a statistical verification of whether navigation performance was related to task order assignment (i.e., to reveal a potential sequence/carry over effect), we also carried out a planned repeated-measures ANOVA. Note that we do not draw any inference regarding task performance based on this latter analysis. Given that this analysis represents an *a priori* determined (i.e., planned) comparison, we did not correct for multiple comparisons. All data was analyzed using SPSS statistical software and no participants were excluded from the analysis.

## RESULTS

All participants were able to interact and explore the virtual environment rendered in AbES under the context of an action video game metaphor. Following game play, assessing behavioral performance suggested that participants were able to transfer the spatial information acquired to the real-world navigation task assessments carried out in the target building. This finding is evidenced by the high rate of success observed in both tasks (see **Table 2**). Specifically, mean success for task 1 (direct route finding) was 70.00% ± 12.91 SD and mean success for task 2 (exit route finding) was 97.14% ± 4.88 SD. For the latter task, mean success in choosing the shortest possible path was 71.43% ± 38.48 SD.

**Table 2:** Summary of navigation task performance.

Task	Measure	Result
Task 1	Mean % correct	70.00% ± 12.91 SD
Task 2	Mean % correct	97.14% ± 4.88 SD
Task 2	Mean % shortest path	71.43% ± 38.48 SD

*Task 1 = direct route finding; Task 2 = exit route finding.*

**FIGURE 3:**
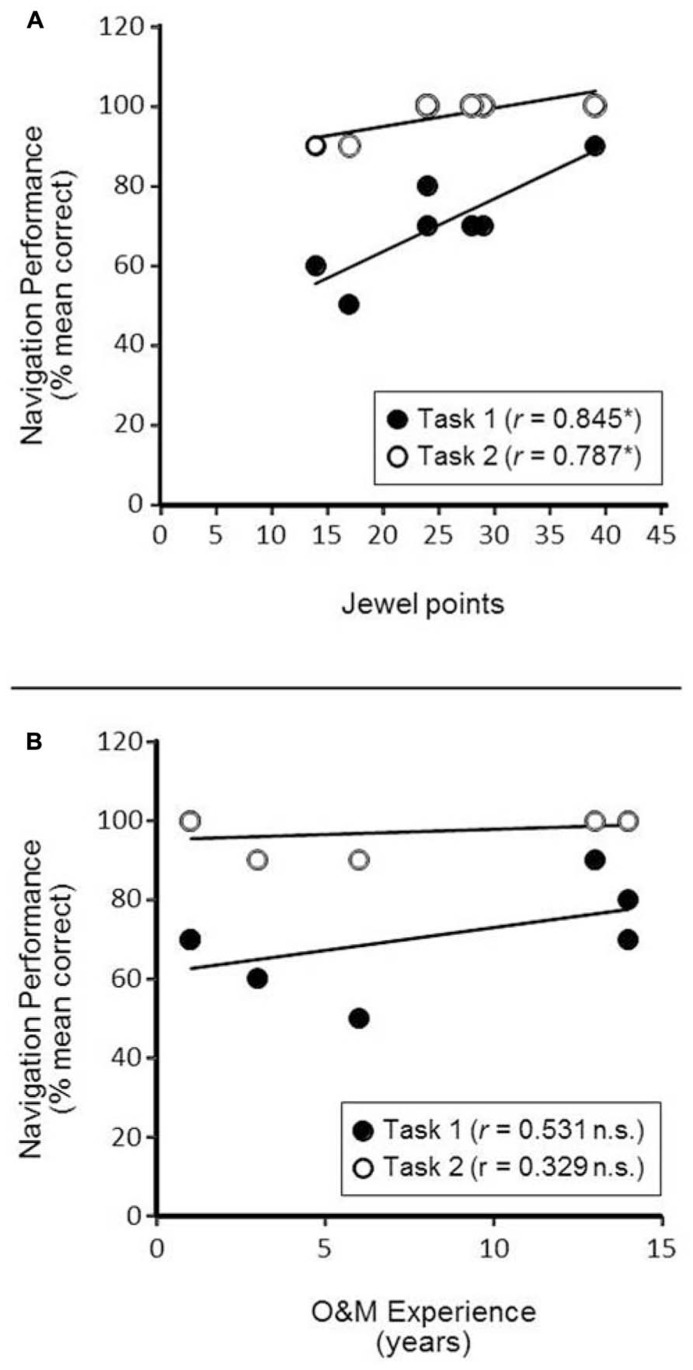
**Correlations between Navigation Task Performance and Factors of Interest.** **(A)** Associating navigation task performance and game play success (number of jewel points) reveals a positive and significant relationship. *p < 0.05 **(B)** Associating navigation performance and O&M experience (measured in years) did not reveal a significant correlation. n.s. = not significant.

We first explored the association between game play success during training on the AbES system (as indexed by the number of jewel points obtained) with overall navigation performance in the building. We found a positive and significant correlation between jewel points obtained and performance in task 1 (direct route finding; mean percentage correct), *r*(5) = 0.845, *p* = 0.017 (**Figure 3A**; filled symbols). A positive and significant correlation between jewel points and performance in task 2 (exit route finding; mean percentage correct) was also evident, *r*(5) = 0.787, *p* = 0.036 (**Figure 3A**; open symbols). The correlation between jewel points and the secondary measure of task 2 performance (mean percentage of shortest paths selected) revealed a positive association but did not achieve statistical significance, *r*(5) = 0.578; *p* = 0.174 (individual data not shown).

As an ancillary analysis, associations between game play performance (scored by raw jewel count) and overall navigation performance confirmed a similar associative trend but did not reach statistical significance [jewels vs. task 1; *r*(5) = 0.722, *p* = 0.067; jewels vs. task 2; *r*(5) = 0.738, *p* = 0.058].

As a control analysis, we next determined the potential association between navigation performance and O&M experience. We re-ran our analysis by comparing individual participant O&M experience (expressed in years of training) with performance on the two navigation tasks of interest and found no significant correlation [O&M vs. task 1; *r*(5) = 0.531, *p* = 0.220; O&M vs. task 2; *r*(5) = 0.329, *p* = 0.471; see **Figure 3B**]. The comparatively low measures of correlation values suggest that navigation performance was not strongly associated with participants’ years of O&M experience.

Finally, we carried out a statistical verification of whether navigation performance was related to task order assignment. For this explicit purpose, a repeated-measures ANOVA was carried out with task (1 and 2) as a within-subjects variable and task order as a between-subjects variable. The results of the ANOVA revealed a significant effect of task [*F*(1,5) = 52.24, *p* < 0.001, $\eta_{p}^{2}$ = 0.913], with better performance for task 2. The main effect of order however, was not significant [*F*(1,5) = 0.126, *p* = 0.737, $\eta_{p}^{2}$ = 0.025] nor was the interaction order by task [*F*(1,5) = 0.816, *p* = 0.408, $\eta_{p}^{2}$ = 0.140]. The lack of effect of task order on the overall navigation performance supports our incorporation of a counterbalanced study design to control for potential carryover/learning effects related to task order.

## DISCUSSION

Audio-based exploration of a large scale indoor virtual environment led to the successful transfer of spatial navigation skills assessed in the target building rendered in the AbES software. Furthermore, through the context of playing an action video game, adolescent participants with early onset blindness were able to accurately acquire and manipulate contextually relevant spatial information and showed a high degree of success on navigation task performance. Finally, performance on the navigation task was positively correlated with game play success. The lack of association between navigation performance and years of O&M training suggests the observed navigation performance was more likely related to the spatial information acquired through game play rather than the participant’s previous level of O&M training and experience.

Overall, these findings suggest that the goal-oriented and self-exploratory nature of AbES provides an engaging, immersive, and safe environment for users to train and develop spatial cognitive skills as they relate to navigation tasks carried out in a corresponding target environment. The encouragement of active exploration and open discovery appeared to promote the generation of spatial mental constructs that could be further manipulated for the purposes of problem solving such as devising novel routes. It is possible that learning the spatial layout through a gaming strategy led to a more robust spatial mental map and subsequently more flexible mental manipulation of the spatial information acquired. This may have in turn led to enhanced contextual learning and transfer of situational knowledge related to a greater understanding of the spatial inter-relations within the building environment.

Interestingly, game play performance was also significantly correlated with real-world navigation task assessment. This latter finding further suggests that the better an individual engages and succeeds in carrying out the exploratory goals of the game metaphor; the better the overall spatial knowledge acquired that is ultimately transferred for the purposes of real-world navigation tasks. This finding is rather striking considering the fact that participants were previously unfamiliar with the layout of the target environment explored and also unaware that their ability to transfer the spatial cognitive information acquired would be tested following game play.

This finding of behavioral enhancement following video game play is in line with recent work investigating the role of video games and spatial cognition in sighted individuals (Green and Bavelier, 2008; Bavelier et al., 2010; Chebat et al., 2011). Mounting evidence has demonstrated that action video game play (in particular, a class of games referred to as “first-person shooter”) leads to changes in spatial attentional processing. Further, changes in sensory and perceptual processing have been shown to lead to demonstrable improvements in spatial cognition (Green and Bavelier, 2003; Spence and Feng, 2010). Video game based learning has also been explored as a means to motivate and engage learners in a variety of contexts including surgical training (Kuppersmith et al., 1996), motor rehabilitation and functional recovery following stroke (Cho et al., 2012), and skill development in children with cognitive and developmental delays such as autism (Strickland, 1997). While the cognitive mechanisms that underlie this form of learning remain the subject of intense investigation (e.g., why certain individuals perform and learn better than others), it is nonetheless intriguing that similar cognitive benefits could also arise from non-visual forms of gaming in the blind. It is possible that the engaging, immersive, and self-paced learning strategy may be more amenable to adolescents who are blind (as opposed to older individuals), who through the rapid development of accessible technology and media are part of the ever expanding population of digital natives. Indeed, the participants in this study were all self-reported experienced and strong users of technology such as smart phones, digital music players and computers.

Despite this encouraging finding, it is important to view these results as preliminary. Obtaining a large sample of individuals as identified in this study represents somewhat of a logistical challenge. From a statistical standpoint, our relatively small sample size represents a potential limitation particularly in terms of carrying out a correlation-based analysis. As such, the magnitudes of the correlations may be as informative as the statistical outcomes for determining significant relationships. To minimize potential confounds, we attempted to enroll individuals that were close in age, blind from early onset, and had similar experience with technology. We also incorporated study features such as a counterbalanced assessment design to minimize task order learning effects. Finally, while we encouraged game play, we were careful to never ask the participants to explicitly memorize the building layout. Despite the relatively high degree of success in task performance observed in this group, a larger scale study is necessary to confirm these findings and further support the potential of this approach as an educative and rehabilitative tool.

How spatial cognitive maps are accurately generated in the absence of prior visual experience (and specifically, for the purposes of complex navigation tasks) raises important questions regarding how the brain creates a mental representation of surrounding space based on available sensory inputs. Further, how spatial information is encoded and ultimately represented when derived from different sensory modalities (e.g., using tactile maps versus verbal instructions) is also an important question that remains unanswered, and clearly has important implications regarding the design of assistive devices for the blind. It is possible that the differences in behavioral performance observed in this study may be related to the method through which spatial information is characterized, the resultant spatial cognitive map generated, and how that spatial information is manipulated. Individuals who are blind have difficulties in identifying the location of distant landmark cues. Thus, the relative contribution and switching between navigation strategies (specifically, egocentric vs. allocentric modes of navigation) may be different from that of sighted individuals. An allocentric reference frame typically describes global (or “survey” level) knowledge of the surrounding environment that is view point independent. In contrast, an egocentric frame characterizes a first-person perspective (or “route” level) and is typically a precursor to developing survey level knowledge (Siegel and White, 1975). It has been suggested that flexible route strategies can arise from survey level knowledge and are important for efficient navigation, particularly in unfamiliar environments (Lederman et al., 1985). With regards to this notion of generating robust and flexible mental spatial representations, related work by Klatzky et al. (2006) has shown that directional information provided by virtual spatial sounds (as opposed to verbal language instructions) help to reduce cognitive load and improve working memory for the purposes of enhancing navigation performance (Klatzky et al., 2006). In this same direction, the advantage of learning a complex spatial layout by interacting with contextual spatialized sounds and within an exploratory game metaphor may further assist in developing more robust mental spatial representations. It would be an intriguing possibility if game based learning approaches could also assist in the development of higher level knowledge and spatial skills beyond the environment explored directly that could ultimately promote greater independence and way finding skills in general. Accounts of children who are blind and who played exploratory video games have documented improved spatial and abstract reasoning as well as social interactions and self-confidence (Sánchez and Lumbreras, 1999; Sánchez and Saenz, 2006; Saenz and Sánchez, 2010). Demonstrating a causal link between video game play and improved general spatial and navigational skills awaits carefully controlled longitudinal studies (i.e., pre–post evaluations) incorporating more developed assessments and metrics of spatial and navigation abilities.

## CONCLUSION

In conclusion, the AbES system facilitated the transfer of spatial knowledge in adolescents who are blind, which allowed them to successfully navigate a real-world building represented in the game. This approach may prove to be a promising step in helping to develop spatial knowledge and real-world navigation skills in individuals who are blind.

## AUTHOR CONTRIBUTIONS

Analyzed the data: Erin C. Connors, Elizabeth R. Chrastil, Lotfi B. Merabet. Designed the research: Lotfi B. Merabet. Collected data: Erin C. Connors, Lotfi B. Merabet. Contributed to writing the paper: Erin C. Connors, Elizabeth R. Chrastil, Jaime Sánchez, Lotfi B. Merabet.

## Conflict of Interest Statement

The authors declare that the research was conducted in the absence of any commercial or financial relationships that could be construed as a potential conflict of interest.
